# Osteopathic Otolaryngology Residency Match Trends: Impact of the ACGME Single Accreditation System (2010–2024)

**DOI:** 10.51894/001c.154562

**Published:** 2025-12-31

**Authors:** Luke Reardon, Brandon Knight, Olga J. Santiago-Rivera, Carl Shermetaro

**Affiliations:** 1 College of Osteopathic Medicine Lincoln Memorial University-DeBusk, Harrogate, Tennessee, USA; 2 Department Otolaryngology-Head and Neck Surgery Residency Program McLaren Oakland Hospital, Pontiac, Michigan, USA; 3 Graduate Medical Education McLaren Oakland, Pontiac, USA

**Keywords:** Otolaryngology, Osteopathic Medicine, Single Accreditation System, Residency Match, Graduate Medical Education.

## Abstract

**INTRODUCTION:**

The 2020 merger creating the Single Accreditation System (SAS) unified the graduate medical education accreditation process under the Accreditation Council for Graduate Medical Education (ACGME). Prior to this merger, osteopathic (DO) and allopathic (MD) residency pathways were separate, particularly limiting osteopathic representation in competitive specialties such as otolaryngology. The impact of the SAS merger on osteopathic applicants matching into otolaryngology residencies has not been quantitatively evaluated. The objective was to evaluate osteopathic applicant trends in otolaryngology residency matches across three distinct periods: pre-merger (2010–2014), transition (2015–2019), and post-merger (2020–2024), surrounding the implementation of the ACGME Single Accreditation System.

**METHODS:**

A cross-sectional analysis was conducted utilizing publicly available residency match data from the National Resident Matching Program (NRMP), National Matching Services (NMS), Accreditation Council for Graduate Medical Education (ACGME), and Fellowship and Residency Electronic Interactive Database (FREIDA). Annual match outcomes were analyzed across three distinct periods: pre-merger (2010–2014), transition (2015–2019), and post-merger (2020–2024). Descriptive statistics and Spearman rank correlation (α = 0.05) were used to evaluate match trends, including the association between calendar year and the number of osteopathic senior matches into ACGME otolaryngology residency programs.

**RESULTS:**

Prior to the merger, during the 2010–2014 period, osteopathic seniors rarely matched into ACGME otolaryngology residencies (average <1 per year), predominantly utilizing the AOA-specific match system (NMS) averaging 22 matches annually. During the transition period, (2015–2019), osteopathic matches slightly increased to an average of four per year. During and following the full implementation of the SAS (2020–2024), osteopathic matches significantly increased from 17 in 2020 to 26 in 2024 (53% increase, p=0.013). Spearman’s rank correlation indicated a strong positive association between year and number of osteopathic matches (ρ=0.915, p<0.001). Allopathic match numbers remained stable (p=0.215), indicating no negative impact on MD candidates.

**CONCLUSIONS:**

The ACGME Single Accreditation System merger was associated with a substantial increase in osteopathic match outcomes in otolaryngology residency programs without an observable decline in allopathic match outcomes. These findings suggest the unified accreditation system expanded access and opportunities for osteopathic medical graduates. These findings have important implications for osteopathic applicant access, match equity, and ongoing GME policy discussions. Future research should investigate specific applicant characteristics influencing match success and program directors’ selection criteria post-merger.

## INTRODUCTION

The completion of the Single Accreditation System (SAS) merger between the Accreditation Council for Graduate Medical Education (ACGME) and the American Osteopathic Association (AOA) in 2020 marked a pivotal moment in the United States graduate medical education (GME). The merger required AOA programs to seek ACGME accreditation, ensuring that residency programs adhere to a common set of standards. This shift also led to a consolidated application timeline and application process, with most specialties participating in the National Resident Matching Program (NRMP), including otolaryngology.^1,2^

Before the SAS merger, osteopathic applicants faced significant challenges securing otolaryngology residency positions. Most DO applicants participated in the National Matching Services (NMS) match under the American Osteopathic Association, which occurred earlier in the application cycle. Between 2010 and 2014, publicly available NRMP data indicated that very few osteopathic seniors matched into ACGME-accredited otolaryngology programs, though the total number of DO applicants was not reported. Many osteopathic applicants withdrew from the NRMP match after securing a position through the AOA match, reducing their representation in ACGME otolaryngology programs. However, no prior study has quantitatively evaluated osteopathic match trends in otolaryngology before and after implementation of the Single Accreditation System.

Osteopathic students faced barriers in pursuing otolaryngology residencies, primarily due to limited program availability in the AOA system, predominant allopathic mentorship in allopathic programs, and fewer resources and research networks than their allopathic counterparts.^3–5^ Additionally, osteopathic applicants were often required to take both the United States Medical Licensing Examination (USMLE) Step 1 and Step 2 and the Comprehensive Osteopathic Medical Licensing Examination of the United States (COMLEX-USA) Level 1 and Level 2. The financial and mental burden of taking both exams and the competitive nature of ACGME programs historically pressured osteopathic students towards the NMS programs.^6^ Recent studies published between 2021 and 2023 have continued to document disparities and structural barriers affecting osteopathic applicants in competitive surgical specialties, including otolaryngology, underscoring the importance of evaluating post-SAS trends.^5,7^

During the SAS transition period (2015–2019), the application landscape for osteopathic candidates became more complex. Over those five years, 26% of all osteopathic surgical residencies either did not apply or voluntarily withdrew their accreditation application.^6^ Of the 21 AOA-accredited otolaryngology programs, 15 initially applied and 13 achieved ACGME accreditation, amounting to 18 residency spots annually. Therefore, 38% (8 programs) did not transition to ACGME accreditation and were ultimately dissolved.^8^ With the elimination of the NMS match, osteopathic students competed directly with allopathic applicants for residency spots. This raised concerns about match rates and long-term opportunities for osteopathic trainees.^6^ A 2023 study examining match rates for surgical subspecialties identified lower match rates for osteopathic applicants, including in otolaryngology, where the osteopathic match rate was 53.1%, compared to 72.6% for allopathic applicants.^7^ Despite these challenges, the SAS merger provided osteopathic applicants easier access to applying to the former AOA and ACGME otolaryngology training programs simultaneously.^2,7^ These contrasting dynamics highlight the post-merger landscape.

This study aims to evaluate the impact of the SAS merger on osteopathic applicants in otolaryngology residency programs, addressing a gap in the literature where no prior publication has analyzed osteopathic match trends over this period. This study aims to evaluate the impact of the SAS merger on osteopathic applicants in otolaryngology residency programs, addressing a gap in the literature where no prior publication has analyzed osteopathic match trends over this period. We hypothesized that implementation of the Single Accreditation System increased osteopathic representation in otolaryngology residency programs. Because this analysis focuses solely on otolaryngology, the findings should not be generalized to all residency specialties or to the broader DO-MD match landscape.

## METHODS

This cross-sectional study examines osteopathic and allopathic applicant match trends in the US otolaryngology residency programs between 2010-2024. Inclusion criteria were an osteopathic otolaryngology residency match. These data were collected from public reports available on the NRMP and NMS website.^9–14^ Extracted data included the number of otolaryngology residency positions, applicants earned degree, and matched applicants earned degree to otolaryngology residency yearly. Applicants were categorized as osteopathic or allopathic based on the degree listed in NRMP and NMS reports (DO vs MD). Former AOA-accredited otolaryngology residency programs were identified using the ACGME Single Accreditation System transition reports, which list all programs that held prior AOA accreditation. Data prior to the merger was limited as the number of osteopathic applicants was not listed. Previously AOA-accredited residencies were identified using the single accreditation system report on the ACGME website.^15^ For post-merger data, program websites for previously AOA-accredited residencies were individually reviewed to collect data on each resident’s medical degree type (osteopathic or allopathic), residency year, and medical school attended. This process was repeated for all accredited otolaryngology residency programs listed in the American Medical Association’s residency database and Fellowship and Residency Electronic Interactive Database (FREIDA) with osteopathic resident composition of at least 1%. The annually updated and publicly accessible websites of the accredited programs were individually searched to identify each resident’s degree, residency year, and medical school attended. Exclusion criteria included training programs lacking publicly available data, websites with incomplete current resident medical education, and osteopathic applicants who matched post-senior year.

*Data Analysis.* All data were imported into Microsoft Excel and R-Studio for analysis. Pre-merger data (2010–2014) were examined, focusing on AOA and ACGME outcomes. Transition period data (2015–2019) were then reviewed to track changes as osteopathic programs sought ACGME accreditation. Finally, merger/post-merger data (2020–2024) were analyzed to assess the impact of a unified accreditation system. These time periods reflect key phases of the ACGME SAS: the pre-merger era of dual accreditation, the transition phase of AOA program integration, and the post-merger period beginning in 2020 with full implementation. Descriptive statistics included frequencies, averages, and percentages. A Spearman’s rank correlation was computed to evaluate the strength and direction of the relationship between calendar year (2010–2024) and the number of osteopathic senior matches into ACGME otolaryngology. A p-value < 0.05 was considered statistically significant.

All statistical analyses were conducted using R within RStudio (version 2024.04.1+748). Spearman rank correlation was selected because annual osteopathic match counts were non-normally distributed and calendar year is an ordinal variable, making a nonparametric measure of monotonic association appropriate. Descriptive statistics were used to summarize the number of residency positions, applicants, and matched applicants across the three study periods.

**Figure 1.**
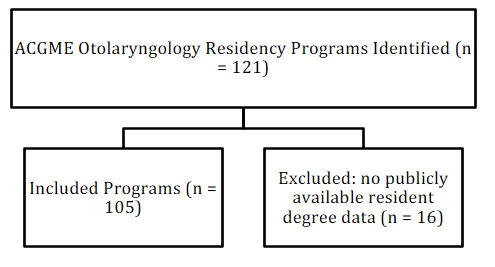
Inclusion and Exclusion Flow Diagram for Residency Programs

**Figure 2.**
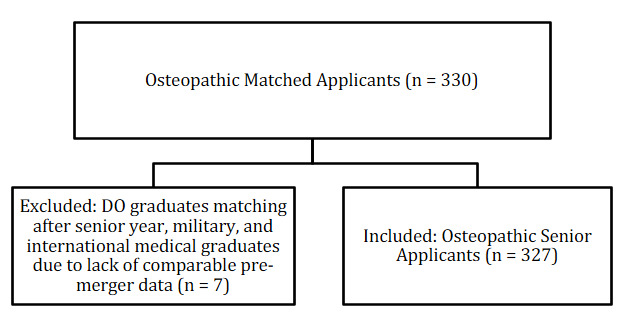
Inclusion and Exclusion Flow Diagram for Osteopathic Matched Applicants

A flow diagram summarizing residency program inclusion and exclusion criteria was created (Figure 1). A second diagram illustrates the inclusion and exclusion of osteopathic matched applicants across the study period (Figure 2). A total of 121 ACGME otolaryngology residency programs were screened. Of these, 16 programs were excluded due to unavailable or incomplete resident degree data, leaving 105 programs in the final analysis. All NRMP-reported osteopathic senior applicants from 2010–2024 were included, while osteopathic graduates who matched after their senior year (n = 7) were excluded from trend analyses due to lack of comparable pre-merger data.

The completeness of available data varied across the study period. NRMP reports did not publish osteopathic applicant counts prior to 2020, preventing calculation of DO-specific match rates for earlier years. Additionally, some residency program websites lacked full resident rosters or degree information, which limited verification for a subset of programs. Applicant-level variables such as board scores, research productivity, demographics, and withdrawal or reapplication status were not available in any public dataset, preventing deeper stratified analysis.

This study was determined to be Not Human Subjects Research by the Lincoln Memorial University IRB and approved by the Scholarly Activity Research Committee at McLaren Oakland. Since only de-identified, publicly available data from NRMP reports were used, no further IRB approval was required.

## RESULTS

Seven individuals, including osteopathic graduates who matched after their senior year of medical school, as well as military and international medical graduates, were identified as matching into ACGME otolaryngology residency programs. However, due to their small sample size, these subjects were excluded from the study as their numbers were insufficient to generate statistically meaningful trends.

**Figure 3.**
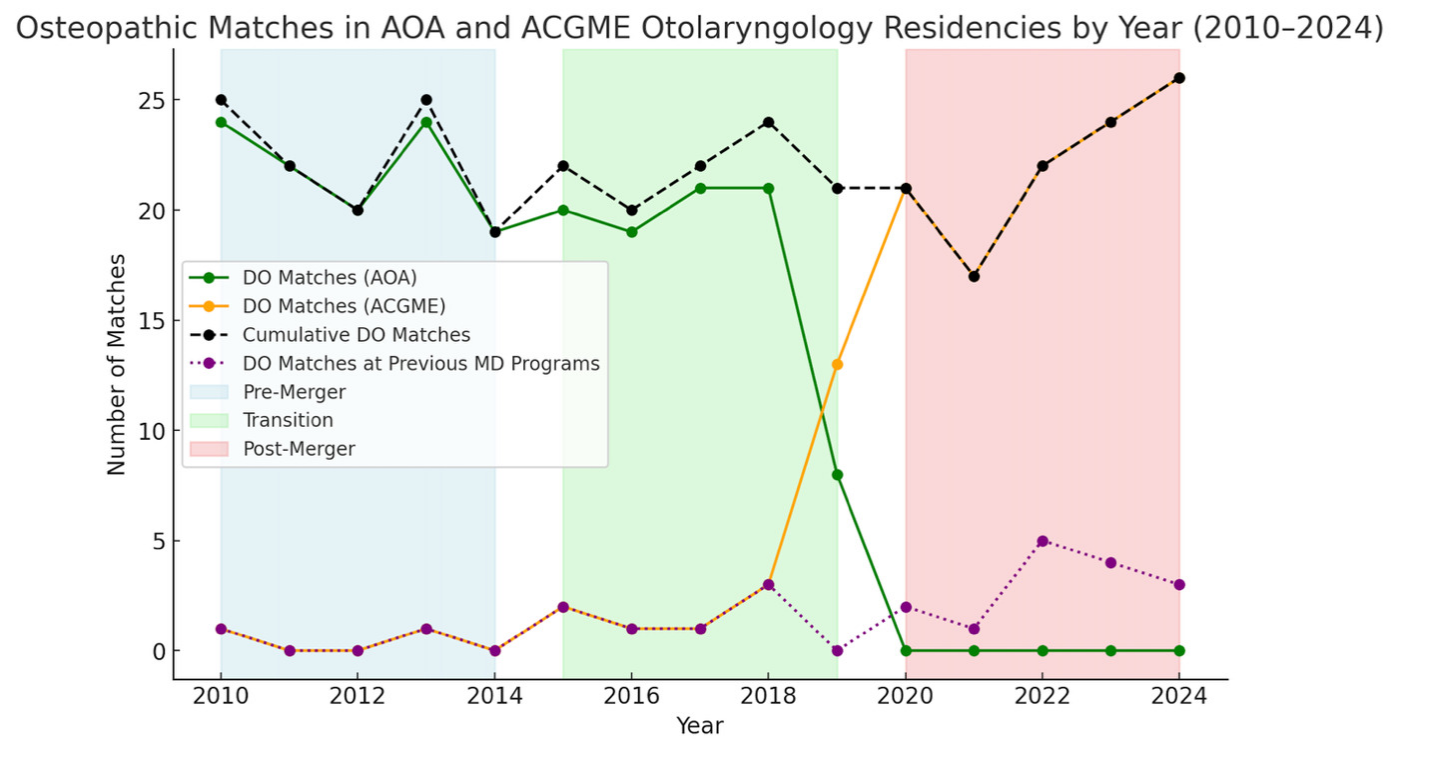
Osteopathic Matches in AOA and ACGME Otolaryngology Residencies by Year (2010–2024)

From 2010 to 2014, ACGME otolaryngology programs offered approximately 280-295 positions, which were predominantly filled by allopathic seniors (around 270 matches per year). Over these five years, only two osteopathic seniors matched into ACGME otolaryngology programs, while the AOA provided 14-18 osteopathic programs annually participating in the NMS match, resulting in approximately 19-24 osteopathic matches each cycle, (Figure 3).

*Number of spots available and applicants.* As shown in Table 1, there was a continuous increase in the number of NRMP otolaryngology residency spots available from the pre-merge period (average 287) to the transition (average 315), and until the end of 2024 (average 363). However, the number and percentage of MD applicants fluctuated each year without an apparent trend from 2010 to 2018. At the end of the transition period in 2019, there was an increase of almost 100 additional applications than the previous year (2018), a growth that continued until 2022, dropped considerably in 2023 (from 463 to 379), but increased again in 2024 (from 379 to 422).

**Table 1.** Distribution of Residency Spots, Applicants, and Matches by Year and Degree Category (MD and DO) for Otolaryngology (2010–2024)

Period		NRMP Spots	MD NRMP Applicants	MD NRMP Matches (Percent of applicants matched)	DO Applicants	DO NMRP Matches (Percent of applicants matched)	AOA Programs	DO AOA ENT Matches
Pre-Merge	2010	280	335	259 (77%)	nd	1	17	24
	2011	283	323	269 (83%)		0	17	22
	2012	285	342	277 (81%)		0	14	20
	2013	292	378	276 (73%)		1	18	24
	2014	295	376	279 (74%)		0	14	19
Pre-Merge 2010-2014 Average		*287*	*351*	*272 (78%)*				
Transition	2015	299	375	283 (75%)		2	15	20
	2016	304	313	272 (87%)		1	13	19
	2017	305	303	279 (92%)		1	15	21
	2018	315	299	284 (95%)		3	13	21
	2019 ++	350	398	308 (77%)		13	6	8
Transition 2015-2019 Average		*315*	*338*	*285 (85%)*				
During & Post-Merge	2020	350	421	310 (74%)	33	17 (52%)		
	2021	350	454	310 (68%)	37	16 (43%)		
	2022	361	463	316 (68%)	41	21 (51%)		
	2023	373	379	310 (82%)	34	23 (68%)		
	2024	382	422	339 (80%)	43	26 (60%)		
Post-Merge 2020-2024 Average		*363*	*428*	*317 (74%)*	*38*	*21* (*55%)*		

*^a^ p* < .05 nd = No data from 2010 to 2019.

++ AOA programs achieved ACGME accreditation in 2019.

This table shows the total number of NRMP positions, applicants, matches (MD and DO), AOA programs, and DO otolaryngology matches under the former AOA system (pre-merger).

*Abbreviations:* NRMP, National Resident Matching Program; AOA, American Osteopathic Association; nd, no data available for that year.

During the transition period (2015–2019), the number of ACGME otolaryngology spots increased from 299 to 350 (Table 1). Allopathic senior matches fluctuated, from 283 of 375 applicants (75%) in 2015 to 284 of 299 applicants (95%) in 2018. However, osteopathic senior matches into ACGME otolaryngology remained low, as depicted in Figure 3. Over these 5 years, the average was four per year. From 2015-2018, no more than three osteopathic applicants matched into an ACGME program, but as AOA programs achieved ACGME accreditation in 2019, this increased to 13 osteopathic applicant ACGME matches. Meanwhile, the AOA match gradually dwindled as programs moved toward or decided to not pursue ACGME accreditation. These changes reflected a combination of formal transitions of AOA-accredited programs into ACGME accreditation, permanent program closures among those that did not pursue accreditation, and broader structural consolidation of otolaryngology training under the unified accreditation system. At the end of the transition, 8 AOA programs had closed, and 13 AOA programs achieved initial accreditation, thus 18 formerly osteopathic residency spots survived.

**Figure 4.**
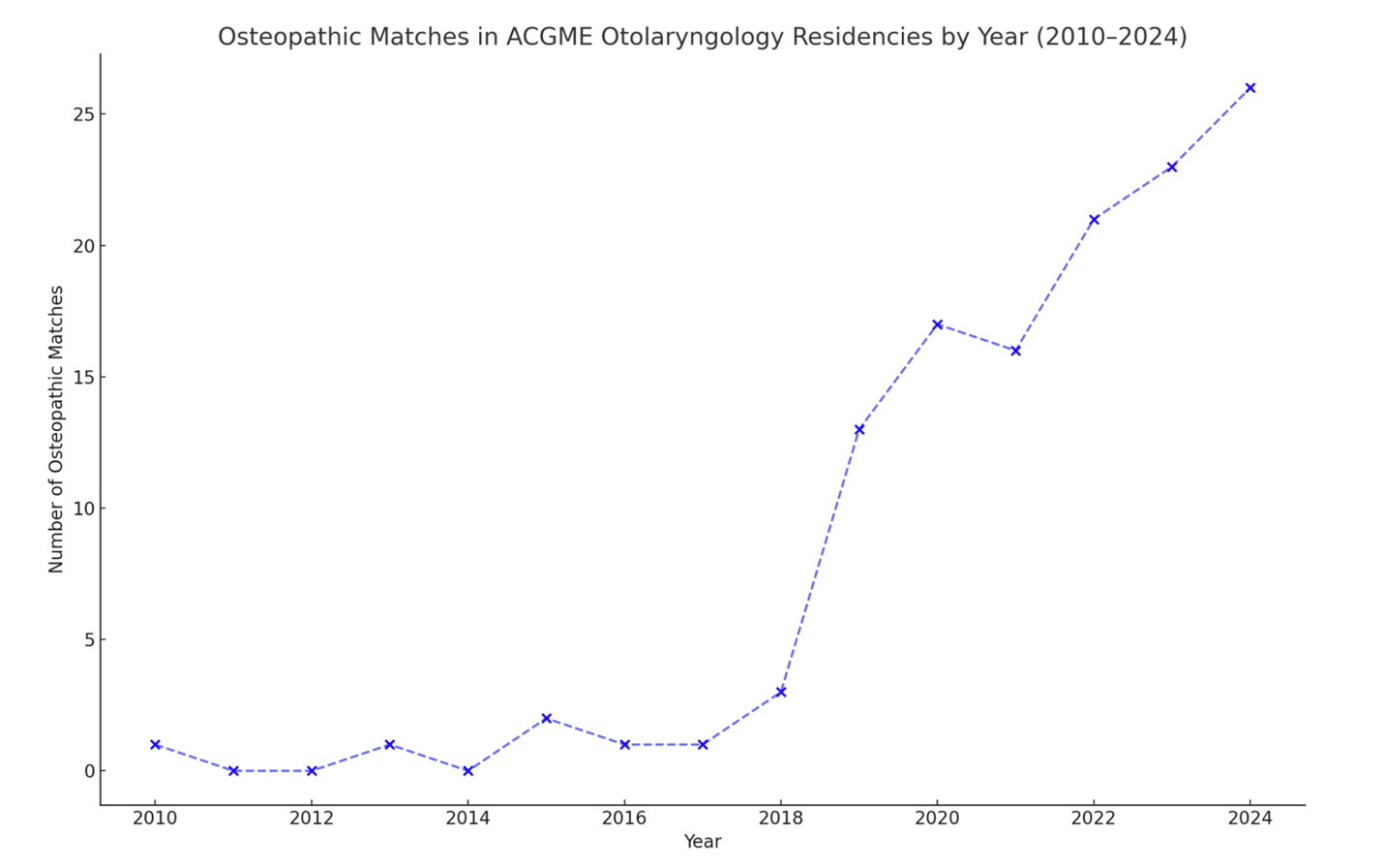
Osteopathic Matches in ACGME Otolaryngology Residencies by Year (2010–2024)

During and following the full implementation of the SAS (Table 1) from 2020 to 2024, there was a notable growth of ACGME accredited otolaryngology spots resulting in a total of 382 available positions in 2024. Osteopathic senior applicants in otolaryngology rose from 33 in 2020 to 43 in 2024 (30% increase), and osteopathic senior matches increased from 17 in 2020 to 26 in 2024 (53% increase, p = 0.013) as shown in Figure 4. A strong positive correlation was found between osteopathic matches and calendar year from 2010 to 2024 (ρ = 0.915, 95% CI 0.758-0.972, p < 0.001), indicating a significant upward trend. This finding suggests that as time progressed from the pre-merger through the post-merger era, the number of osteopathic matches consistently and significantly trended upward. Allopathic match numbers remained relatively stable from 2020-2024, with no statistically significant change (p = 0.215; Table 1). The highest number of osteopathic residents matched in one year occurred in 2024 at 26 matches, surpassing all prior annual totals (Figure 3). Likewise, the largest group of osteopathic residents in a historically allopathic residency setting was also observed post-merger, peaking in 2022.

After reviewing public residency websites, 19 originally allopathic residency spots were filled by osteopathic applicants in 16 different programs over the years 2020 to 2024.^16–25^ This is drastically more than the two osteopathic matches in the NRMP during the five years before the merger was initiated. The 13 original AOA programs that obtained ACGME single accreditation amounted to 92 residency spots and since 2020, four (4.3%) spots were filled by allopathic applicants with the remaining filled by osteopathic applicants (n=88, 95.7%). At least one otolaryngology military residency spot was filled by an osteopathic applicant over this timeframe.

## DISCUSSION

The results of this study demonstrate a significant upward association between calendar year and the number of osteopathic applicants matching into ACGME-accredited otolaryngology residency programs in the post-merger period. Historically, from 2010 to 2014, osteopathic candidates faced limited representation restricted by dual accreditation pathways and limited positions. While the transition period (2015–2019) saw some improvement, osteopathic matches into ACGME otolaryngology programs remained relatively low. A significant upward trend in osteopathic matches was observed during the period following implementation of the merger in 2020.

Similar trends have been observed across other competitive surgical and perioperative specialties following implementation of the Single Accreditation System. In general surgery, multiple studies have demonstrated increased osteopathic participation in ACGME-accredited training programs after unification of the match systems. In neurosurgery, although osteopathic applicants continue to face substantial barriers, recent analyses suggest gradual improvement in representation following the merger. Likewise, in anesthesiology, a specialty that transitioned rapidly into the unified match, osteopathic match participation has expanded steadily since 2020. These cross-specialty findings suggest that the improved osteopathic match outcomes observed in otolaryngology may reflect a broader, system-wide restructuring effect of the Single Accreditation System, rather than an isolated, specialty-specific phenomenon.

Our findings reveal a strong positive correlation (ρ = 0.915, p < 0.001) between calendar year and osteopathic matches. This positive correlation may reflect an increase in osteopathic graduates, greater program director familiarity with osteopathic applicants, or an expanded number of total available positions. Before 2014, most osteopathic applicants used the NMS match system. The integration of the match systems occurred alongside increased access for osteopathic applicants, though this temporal association alone does not fully explain the rise in osteopathic match success. The increase is also consistent with a larger pool of osteopathic medical students, which grew from 5,263 graduates in 2014 to 8,200 in 2024, greater exposure of programs to osteopathic applicants through rotations, and potentially improved osteopathic application quality in response to the unified, competitive landscape.^26–28^ Additionally, osteopathic applicants may have gained increased exposure to allopathic programs during the transition period as they anticipated the merger, allowing program directors to familiarize themselves with these candidates. It is also possible that some programs may value the diversity brought by osteopathic applicants’ educational backgrounds.^29,30^ Because this was a retrospective observational analysis using aggregate, program-level data, these findings demonstrate associations and temporal patterns rather than causal effects of the Single Accreditation System on match outcomes.

Institutional and regional variation may also influence osteopathic representation in otolaryngology residency programs. Differences between large academic medical centers and community-based or hybrid programs in terms of clinical focus, research expectations, and historical exposure to osteopathic training may affect applicant selection patterns. Because this study did not systematically collect program-level characteristics such as academic affiliation, community status, or geographic region, we are unable to determine whether these structural factors contributed to the observed national trends. Future research should evaluate whether osteopathic match outcomes differ meaningfully by program type or region.

While osteopathic match numbers have shown an increasing trend, allopathic match rates remained stable from 2020 to 2024, suggesting that increasing osteopathic representation was not associated with a decline in domestic allopathic student match chances during this period.

While this study focused on osteopathic seniors matching into ACGME otolaryngology programs, seven osteopathic graduates obtained positions after their senior year during the SAS post-merger period. This would increase the total osteopathic otolaryngology residency post-merger matches from 103 to 110. These individuals were excluded from the primary analysis to maintain statistical validity as osteopathic graduate match data was not listed before 2020. Their exclusion does not diminish their significance but suggests the need for a larger dataset to assess trends within this subgroup.

A transient decline in both osteopathic and allopathic otolaryngology applicants was observed in 2023, with osteopathic applicants decreasing from 41 to 34 and allopathic applicants decreasing from 463 to 379. This decrease did not follow the application stability observed from 2020 to 2022. Prior otolaryngology match analyses have demonstrated cyclical fluctuations in applicant volume over time, suggesting that changes in perceived competitiveness or prior-year match outcomes may influence applicant behavior rather than structural accreditation changes alone.^31^

This study has several important limitations. First, the analysis is restricted to a single specialty, otolaryngology, and therefore the findings should not be generalized to other residency specialties or to the broader Osteopathic-Allopathic match landscape. Second, the study relied exclusively on publicly available NRMP, NMS, and program website data, which do not capture unmatched applicants, withdrawn applicants, or applicants who reapplied in subsequent cycles. Third, several applicant-level variables were unavailable, including board examination scores, research productivity, demographics, and detailed academic performance metrics, limiting risk-adjusted or predictive analysis. Finally, incomplete or outdated residency website data may have resulted in under-ascertainment of osteopathic representation at a small number of programs.

This study opens avenues for future research. Future studies could investigate applicant-specific factors such as board scores, research publications, personal characteristics (e.g., race, ethnicity, sex), and clinical rotations. Additionally, research into shifts in program directors’ perceptions or ranking strategies could provide further insight into the increased acceptance of osteopathic applicants. Investigating other competitive specialties could also provide further understanding of the merger’s impact on osteopathic applicants, and tracking osteopathic applicants over time could show long-term trends and outcomes.

## CONCLUSION

In conclusion, this study demonstrates that osteopathic representation in otolaryngology residency programs increased during the post-Single Accreditation System era, with important implications for graduate medical education access, equity, and workforce development. At the same time, stable allopathic match rates indicate that unified accreditation has not negatively impacted allopathic applicants. Future research should include continued longitudinal tracking of osteopathic applicant access to otolaryngology and other surgical subspecialties beyond 2024 to determine whether these post-merger trends are sustained over time. Ongoing evaluation of program-level and applicant-level factors will be essential for informing educational policy and ensuring equitable access across competitive surgical training pathways.
